# Case Report: Development of persistent genital arousal disorder due to a possible combination of medications

**DOI:** 10.3389/fpsyt.2025.1709728

**Published:** 2025-11-25

**Authors:** Ludek Fiala, Jiří Lenz

**Affiliations:** 1 Psychiatric Clinic, Department of Sexology, Faculty of Medicine, Charles University, Pilsen, Czechia; 2 Institute of Sexology, First Faculty of Medicine, Charles University, Prague, Czechia; 3 Department of Pathology, Znojmo Hospital, Znojmo, Czechia; 4 Department of Anatomy, Histology and Embryology, Faculty of Veterinary Medicine, University of Veterinary and Pharmaceutical Sciences Brno, Brno, Czechia

**Keywords:** persistent genital arousal disorder, menopause, trazodone, pregabalin, estriol, medroxyprogesterone, pharmacological interaction

## Abstract

**Objective:**

Persistent Genital Arousal Disorder (PGAD) is a rare and distressing condition characterized by persistent genital arousal in the absence of sexual desire. We present a unique case of a menopausal woman who developed PGAD after combined pharmacotherapy with trazodone, pregabalin, vaginal estriol, and depot medroxyprogesterone acetate (DMPA).

**Methods:**

The patient underwent comprehensive gynecological, neurological, and psychiatric evaluation. Menopausal status was confirmed by laboratory findings. A detailed pharmacological review was conducted to assess possible interactions affecting serotonergic, neuronal, and hormonal regulation.

**Results:**

PGAD symptoms emerged shortly after initiating the combination of these medications. Each drug has been individually associated with alterations in sexual function, but their concurrent use may have produced synergistic effects on neuroendocrine pathways. Gradual discontinuation of all medications resulted in complete remission of symptoms.

**Conclusion:**

This case supports the hypothesis that combined modulation of serotonergic, neuronal, and hormonal systems can trigger PGAD in predisposed menopausal women. Recognition of potential pharmacological interactions is crucial for diagnosis and management of PGAD.

## Graphical Abstract

Deterioritation of patient´s condition, hospitalized with diagnosis of PGAD.

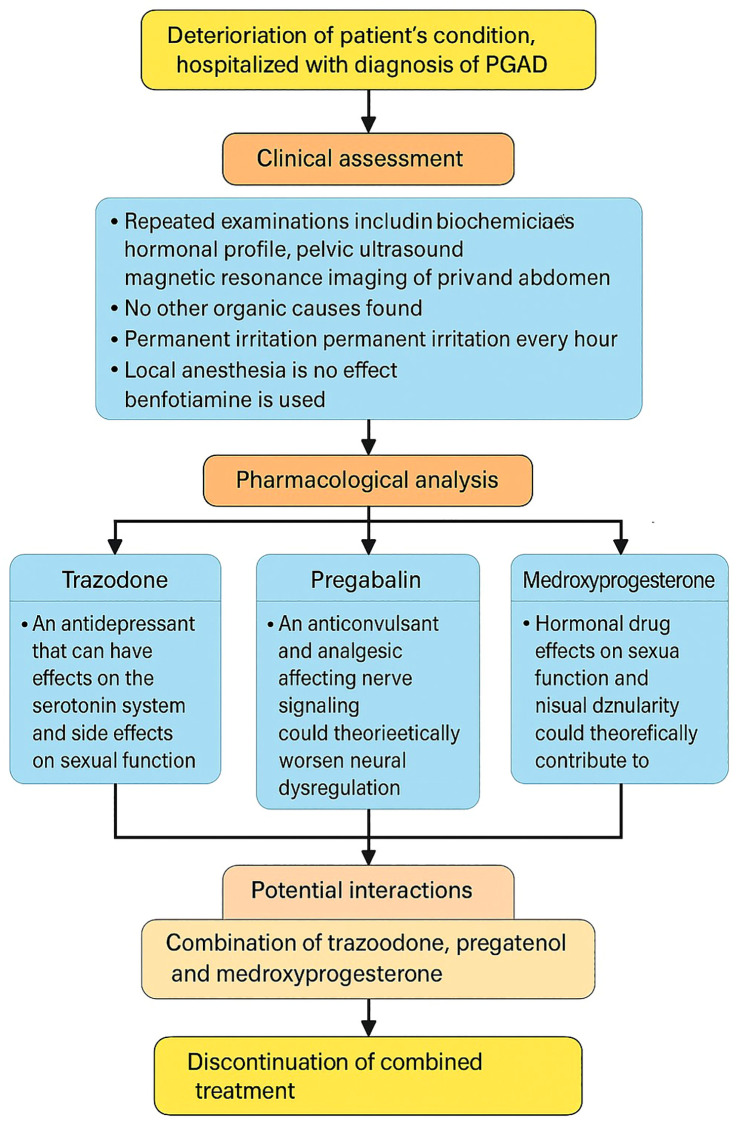

## Introduction

Persistent Genital Arousal Disorder (PGAD) is a rare and complex condition characterized by persistent, unwanted genital arousal in the absence of sexual desire (1). Unlike normal sexual arousal, these sensations are involuntary, intrusive, and often distressing, significantly impairing quality of life and emotional well-being (2).

The prevalence of PGAD is estimated at around 1% of women, though it is likely underdiagnosed due to stigma and limited clinical awareness (1). According to the International Society for the Study of Women’s Sexual Health (ISSWSH), the diagnostic criteria include: (1) persistent, spontaneous genital arousal not associated with sexual interest; (2) symptoms lasting hours or days; (3) incomplete or short-lived relief after orgasm; (4) marked distress or functional impairment; and (5) exclusion of other medical or psychiatric causes (3).

The etiology remains unclear, but multiple interacting factors are implicated, including neurological, hormonal, and pharmacological mechanisms (4, 5). Serotonergic dysregulation, pudendal nerve hypersensitivity, Tarlov cysts, and hormonal fluctuations during menopause have all been proposed as contributing factors. Pharmacological triggers—particularly medications influencing serotonin and hormone pathways—have been reported as potential causes (6).

Menopausal women are particularly vulnerable due to fluctuating hormone receptor sensitivity and altered neuroendocrine balance. This report presents, to our knowledge, the first documented case of PGAD associated with the simultaneous use of trazodone, pregabalin, estriol, and DMPA.

## Case presentation

A 51-year-old menopausal woman presented with newly developed persistent genital arousal symptoms. Laboratory results confirmed menopause (FSH 51 IU/L; estradiol 0.08 nmol/L). She had no prior psychiatric, neurological, or endocrine disease.

Initially, she sought treatment for menopausal symptoms—insomnia, hot flashes, and vaginal discomfort. Gynecological examination and vaginal cultures were normal. She received one injection of depot medroxyprogesterone acetate for irregular bleeding and was prescribed trazodone (50 mg, later 75 mg daily) for sleep. Two weeks later, persistent vaginal irritation led to the addition of estriol cream (1 mg/g, three times weekly). When discomfort persisted, pregabalin was prescribed (150 mg/day, increased to 300 mg/day).

Within three weeks, she developed continuous genital sensations—tingling and throbbing in the clitoral and vaginal areas—unrelated to sexual desire. These caused compulsive masturbation attempts every hour, with no relief. The condition severely disrupted her ability to work and caused distress in her marital relationship.

She was hospitalized for evaluation. Neurological and imaging studies were normal. A multidisciplinary consultation identified a likely pharmacological etiology. A deprescription strategy was implemented: estriol was discontinued first, followed by gradual cessation of pregabalin and trazodone. No further DMPA injections were given. Over three months, symptoms subsided, and by the fourth month, she reported complete remission.

## Discussion

PGAD is thought to arise from complex neuroendocrine dysregulation. This case shows a probable pharmacological trigger involving concurrent serotonergic, neuronal, and hormonal modulation.

Trazodone, a serotonin antagonist and reuptake inhibitor, can alter genital sensory processing via 5-HT2A and 5-HT1A receptors. Although typically associated with priapism in men, it may similarly affect genital blood flow and neural excitation in women (6).

Pregabalin binds to α2δ subunits of voltage-gated calcium channels, reducing neural excitability, but paradoxical hyperexcitability has been described due to central disinhibition (5).

Estriol enhances mucosal blood flow and increases genital sensory receptor density in hypoestrogenic tissue. Medroxyprogesterone acetate modulates dopaminergic and serotonergic systems and may alter neuroendocrine equilibrium (1).

In menopausal women, this combination may create a state of peripheral nerve hypersensitivity and central neurotransmitter imbalance, resulting in persistent genital arousal. While PGAD has been linked separately to serotonergic or hormonal agents, this is the first known case describing the synergistic impact of all four.

### Clinical implications

Clinicians should consider medication-induced PGAD when evaluating unexplained genital arousal symptoms, especially in menopausal women exposed to multiple interacting drugs. Early deprescription and pharmacological review can lead to full recovery and avoid unnecessary invasive investigations.

### Strengths and limitations

The case demonstrates a strong temporal relationship and full reversibility, supporting a causal hypothesis. Limitations include the absence of neurophysiological testing and the single-case design. Nevertheless, the coherence between pharmacological reasoning and clinical outcome provides compelling evidence.

### Future directions

Further research should explore neurochemical biomarkers of PGAD and evaluate pharmacogenetic vulnerability to serotonergic and hormonal interactions in menopausal patients.

### Patient perspective

The patient expressed profound distress during the acute phase of her symptoms, describing a sense of loss of control and social isolation (7). After discontinuation of all medications, she reported complete resolution of symptoms and a return to normal sexual and emotional functioning. She expressed gratitude that the underlying cause was identified and managed without surgical intervention.

## Conclusion

This case illustrates that PGAD may develop through overlapping serotonergic, neuronal, and hormonal mechanisms in biologically vulnerable individuals. The temporal correlation between medication exposure and symptom onset, along with complete remission after deprescription, supports a drug-induced pathogenesis. Awareness of these interactions can improve diagnosis and treatment.

## Data Availability

The original contributions presented in the study are included in the article/supplementary material. Further inquiries can be directed to the corresponding author.
